# Identification and Characterization of AP2/ERF Transcription Factors in Yellow Horn

**DOI:** 10.3390/ijms232314991

**Published:** 2022-11-30

**Authors:** Fang Hu, Yunxiang Zhang, Jinping Guo

**Affiliations:** The College of Forestry, Shanxi Agricultural University, Jinzhong 030801, China

**Keywords:** AP2/ERF, identification, yellow horn, characterization

## Abstract

The AP2/ERF gene family involves numerous plant processes, including growth, development, metabolism, and various plant stress responses. However, several studies have been conducted on the AP2/ERF gene family in yellow horn, a new type of oil woody crop and an essential oil crop in China. According to sequence alignment and phylogenetic analyses, one hundred and forty-five AP2/ERF genes were detected from the yellow horn genome. They were divided into four relatively conserved subfamilies, including 21 AP2 genes, 119 ERBP genes, 4 RAV genes, and 1 Soloist gene. Gene analysis of *XsAP2/ERF* TFs showed 87 *XsAP2/ERF* TFs lacked introns. There were 75 pairs of collinearity relationships between *X. sorbifolium* and *Arabidopsis*, indicating a close similarity. In addition, the expression patterns of *XsAP2/ERF* TFs under cold treatments confirmed that the *XsAP2/ERF* TFs play essential roles in abiotic stress response. The expression of eight *XsAP2/ERF* transcription factors was verified in different tissues and under various stress treatments using RT-qPCR. This study establishes a starting point for further research to explore the potential mechanisms of identifying candidate AP2/ERF TFs that could respond to the abiotic stress of yellow horn.

## 1. Introduction

The increasing frequency of extreme weather and climate events and various other abiotic stresses globally threaten plant growth, health, and productivity. Due to the continuous accumulation of environmental conditions, the survival of plants is facing more and more severe challenges. Plants enhance their tolerance to these stresses through intricate regulatory mechanisms [1]. These mechanisms cause physiological and biochemical responses and developmental changes [2,3]. It is worth noting that plants contain many transcription factors (TFs) that control plant growth, development, and response to environmental stress [4,5,6]. Transcription factors that activate the abscisic acid and ethylene signaling pathways play an essential role in plant responses to abiotic stresses [7] by binding specifically to repressor regions in the promoter regions of downstream target genes. Throughout activating or inhibiting downstream functional genes, cis-acting elements can regulate gene expression [8]. Several transcription factor families have been identified in plants, including AP2/ERF, MYB, HSP, Bzip, and NAC [6]. The AP2/ERF gene family is a large gene group that encodes transcription factors specific to plants [9,10]. These transcription factors are essential for regulating plant gene expression and play a key role in various aspects of plant development [11,12].

The AP2/ERF genes contain 60–70 amino acid AP2/ERF DNA binding domains composed of YRG and RAYD motifs. Based on the number of AP2/ERF domains and the similarity of amino acid sequences, the AP2/ERF TFs can be classified into four categories: (1) The AP2 family contains two AP2 domains; (2) The EREBP family contains one AP2 domain [13], which is further subdivided into ERF and DREB subfamilies depending on sequence similarities [14]; (3) RAV family contains a single AP2 and B3 domain [15], mainly regulated by ethylene or brassinosteroid participates in biotic and abiotic stresses [16,17]; (4) Soloist family contains one AP2 domain and acts as a positive regulator of the disease defense mechanisms [18]. These TFs are involved explicitly in regulating defense responses against various biological stresses and controlling different multiple stress responses, such as pathogen-induced stimuli [19,20,21], drought [22,23], heat [24], and salt stress [23].

With the release of many plant genome reference sequences, research on the AP2/ERF gene family has been conducted in *Arabidopsis* [10], rice [25], grape [26], soybean [27], and poplar [28]. However, no study has explored the identification and characterization of the AP2/ERF family in yellow horn (*Xanthoceras sorbifolia*).

## 2. Results

### 2.1. Identification and Classification of AP2/ERF

This study identified 145 *XsAP2/ERF* TFs using HMM profile searches and blasts in the *X. sorbifolium* genome. The *XsAP2/ERF* genes were classified into AP2, EREBP, RAV, and Soloist. The 21 genes were assigned to the AP2 subfamily, of which 18 had two identical AP2 domains, and 3 contained only one. However, the 119 EREBP TFs were further divided into DREB and ERF branches. According to the sequence similarity, the ERF and DREB branches were categorized into six subgroups (A1 to A6, B1 to B6), respectively. The RAV subfamily (four members) has one AP2/ERF domain and an additional B3-DNA binding domain. The remaining gene was assigned to the Soloist, which showed higher similarity with *At4g13040*. Based on their chromosomes order and physical positions, they were named from *XsAP2/ERF1* to *XsAP2/ERF65*, *XsDREB1* to *XsDREB54*, *XsRAV1* to *XsRAV4* and *XsSoloist1*. More characteristics, including gene IDs, and other features, were listed in Table S1. The molecular weight of the protein ranged from 11.86 kDa to 83.67 kDa, while the isoelectric point varied from 4.6 (*XsERF36*) to 10.38 (*XsERF51*).

### 2.2. Phylogenetic Analysis

**Figure 1:**
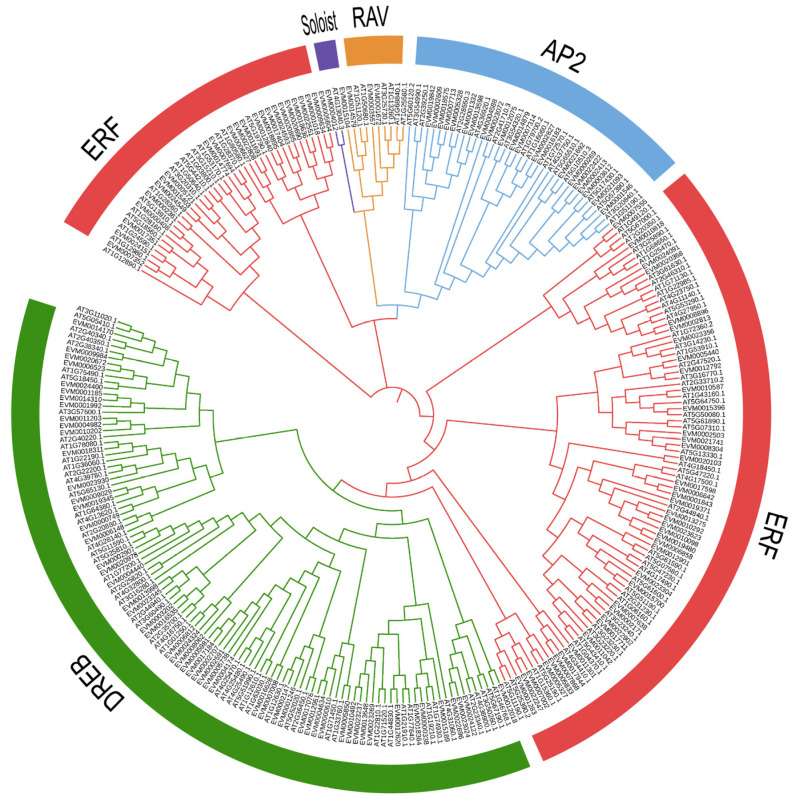
Phylogenetic tree of AP2/ERF genes between *X. sorbifolium* and *A. thalian*. The ERF, DREB, AP2, RAV, and Soloist families are distinguished by different colors.

**Figure 2:**
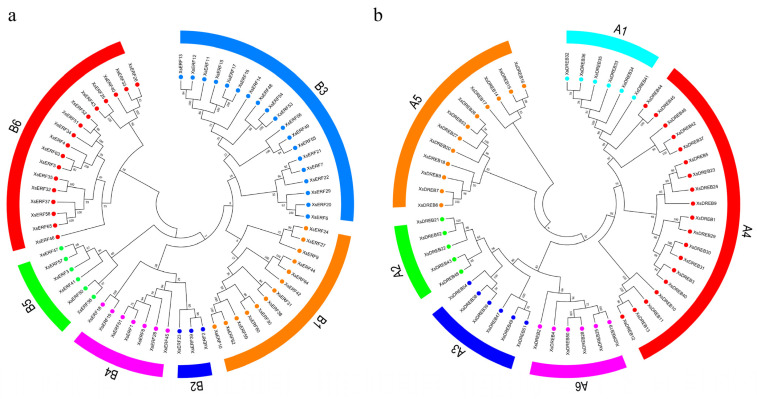
Phylogenetic trees of the ERF and DREB subfamilies of yellow horn. (**a**) Phylogenetic tree of ERF subfamily; (**b**) Phylogenetic trees of subfamilies. The 12 clades of ERF and DREB subfamily genes (B1–6 and A1–6) were grouped depending on their previous classification in *Arabidopsis*.

The phylogenetic relationship of *XsAP2/ERF* genes was investigated using the amino acid sequences of 145 *XsAP2/ERF* from *X. sorbifolium* and 175 *AtAP2/ERFs* from *A. thaliana*. A phylogenetic tree was constructed to reveal the evolutionary history between these genes. As shown in Figure 1, all the *XsAP2/ERFs* were classified into five groups, named DREB, ERF, AP2, RAV, and Soloist. Among them, 56 DREBs were distributed into groups A1- A6, containing 6, 5, 6, 19, 12, and 6 genes, respectively (Figure 2). Additionally, 65 ERFs were distributed into groups B1-B6 containing 13, 3, 19, 7, 6, and 17 genes, respectively. Notably, the ERF subgroup was the largest, whereas the Solosist subgroup was the smallest, with only one member.

### 2.3. Gene Structure and Conserved Motif Analysis

The number of exons and introns and the distribution of conserved domains was investigated to gain insights into the structural diversity of *XsAP2/ERF* genes (Figure S1). We found that the number of introns varied significantly, with 58% of AP2/ERF genes having no introns. The four *XsDREB* genes (2, 34, 35, and 41) had introns ranging from one to four, except *XsDREB* genes contained no introns. A total of 39 ERF genes do not possess introns. Unlike the ERF gene, all 21 AP2 genes contained introns, ranging in number from two to nine. For the four RAV genes, *XsRAV1* had one intron, *XsRAV4* had three introns, and the other two RAV genes had no introns. One Soloist gene has six introns. The intron distribution of the *XsAP2/ERF* gene suggested that the AP2 and Soloist subgroups contained more introns in comparison to the other three subgroups.

All AP2/ERF proteins were investigated by MEME, revealing 25 conserved motifs within the families and subfamilies. Motif-1 was present in all *XsAP2/ERF* proteins. Most of the *XsAP2/ERF* also showed motif-2 and motif-3. As predicted, similar compositions of conserved motifs among closely related members suggest functional similarities within the same subfamily.

### 2.4. Chromosome Distribution and Duplication

**Figure 3:**
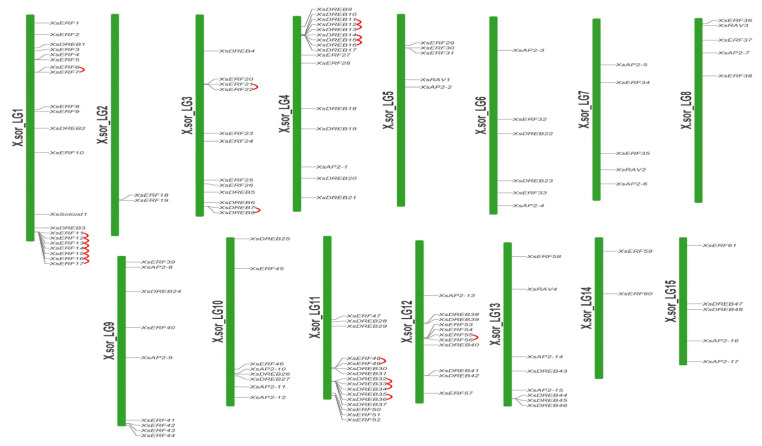
Schematic diagram of AP2/ERF gene chromosome distribution in yellow horn. The *XsAP2/ERF* genes were located on 15 chromosomes, representing gene positions by proportion. A line indicates position of a gene on each chromosome. The red line between the two gene names suggests that they are tandem repeat pairs. Chromosome numbers are represented on the right side of each chromosome.

The locations of genes on chromosomes were generated using the software map chart. The *XsAP2/ERF* genes were renamed according to the chromosomal distribution. There was a great deal of variation in the number of *XsAP2/ERF* genes among chromosomes (Figure 3). For example, chromosome 1 contained the most genes (12), while chromosomes 2 and 14 had only two genes. There was no observed relationship between chromosome length and the distribution of *XsAP2/ERF* genes. Moreover, the Soloist gene was found on chromosome 1, while the *XsRAV* family members were located on chromosomes 5, 7, 8, and 13. The research showed an uneven distribution of *XsAP2/ERF* genes on the chromosomes.

**Figure 4:**
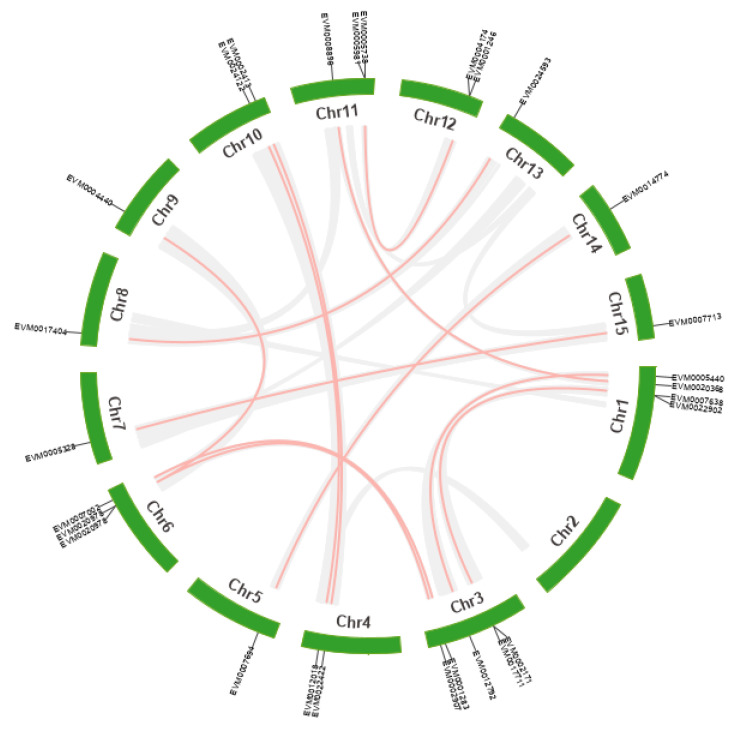
Schematic diagram of the relationship between AP2/ERF TFs in yellow horn. The visualization of all AP2/ERF family repeat gene pairs in chromosome Chr1-15 was performed using Circos. The gray lines in the figure represent collinear blocks, and the red lines represent repeated AP2/ERF gene pairs with collinear relationships in yellow horn.

**Figure 5:**
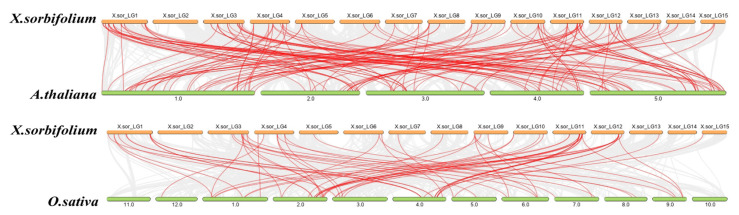
Homolinear AP2/ERF gene analysis between *X. sorbifolium*, rice, and *A. thaliana*. The gray lines in the background indicate colinear blocks within the genomes of *X. sorbifolium* and other plants, while the red line denotes the colinear AP2/ERF gene pairs.

The MCScanX package discovered 18 gene tandem duplication events containing 35 *XsAP2/ERF* genes on chromosomes. Among the tandem repeat genes, eight pairs were DREB genes, and 10 were ERF genes. In addition, 14 segmental duplication events with 27 genes happened, one between alleles and 13 between non-alleles in the yellow horn genome (Figure 4). Two tandem duplication pairs happened in chromosomes 3 and 12, four tandem duplication pairs happened in chromosomes 4 and 11, and chromosome 1 has seven duplicates. Segmental repeat events were also detected in each chromosome (Figure 5). These results suggest that large-scale segmental duplication events are essential to expanding the *XsAP2/ERF* family. To explore the mechanism of *XsAP2/ERF* genes divergence after duplication and examine the selection effect, we calculated the nonsynonymous substitution ratios (Ka) and synonymous substitution ratios (Ks), and nucleotide substitutions (Ka/Ks ratio) for both tandem and segmented duplications (Table S2). The Ka/Ks ratio for tandem repeat genes in the AP2/ERF TFs ranged from 0.17 to 0.72. This suggests that all genes within this family are evolving under negative selection pressure.

The evolutionary origins of the AP2/ERF gene in yellow horn could be examined by constructing semilinear maps of two representative species. A comparison of direct homologous gene pairs across all chromosomes showed that 75 *XsAP2/ERF* genes were synonymous with *A. thaliana* genes, and 39 *XsAP2/ERF* genes were synonymous with rice genes.

### 2.5. Analysis of Cis-Regulatory Elements (CRE)

Cis-regulatory elements (CREs) play a vital role in genetic regulatory networks because of the DNA sequences they contain in non-coding regions. Transcription factors usually bind to the CREs region to control the transcription and expression of neighboring genes. Using the PlantCare tool, we could scan the cis-acting regulatory elements in the 2000-bp sequence upstream of the translation start site of *XsAP2/ERF* genes. The statistical results for the cis-acting elements showed that the regulation of *XsAP2/ERF* genes is mainly associated with MeJA, ABA, IAA, GA, and SA (Figure S2). In addition to the transcription factors, cis-regulatory elements were related to various stress responses, such as wound healing, defense, stress tolerance, hypoxia, anaerobic, and hypothermia.

In this study, a total of 119 *XsAP2/ERF* genes were found to contain abscisic acid response elements, 93 genes contained gibberellin response elements, and 101 genes contained MeJA response elements. Another 37 *XsAP2/ERF* genes contained low-temperature responsive elements, and 145 contained light-responsive elements. Soloist genes tend to have fewer CREs than other genes linked to hormones and stress responses. Although the five gene families shared similarities in their CREs types, the number of each CRE was different. The analyses of cis-regulatory elements suggest that *XsAP2/ERF* is essential for plant development and adaptation to environmental stress conditions.

### 2.6. Expression Pattern

#### 2.6.1. The Expression Using RNA-Seq

To understand the expression pattern of *XsAP2/ERF* genes, we analyzed the expression profiles of the identified genes under salt, alkali, and low-temperature stress using RNA-seq data. Heat maps of *XsAP2/ERF* gene expression profiles in leaves were drawn by TBtools (Figure S3).

Twenty-nine *XsAP2/ERF* genes were expressed under cold stress. We found nine *XsAP2/ERF* genes were up-regulated, and one gene was down-regulated under low-temperature stress for 4 h. There were 19 up-regulated and three down-regulated expressions under low-temperature stress for 12 h. Under low-temperature stress for 24 h, there were 15 up-regulated and three down-regulated expressions. Among them, the co-expressed six *XsAP2/ERF* genes (*XsDREB20/32/33/48*, *XsERF30/45*) were up-regulated and included in all treatments. However, none of the *XsAP2/ERF* genes were differentially expressed under salt and alkali stress.

#### 2.6.2. The Expression Levels by RT-qPCR

**Figure 6:**
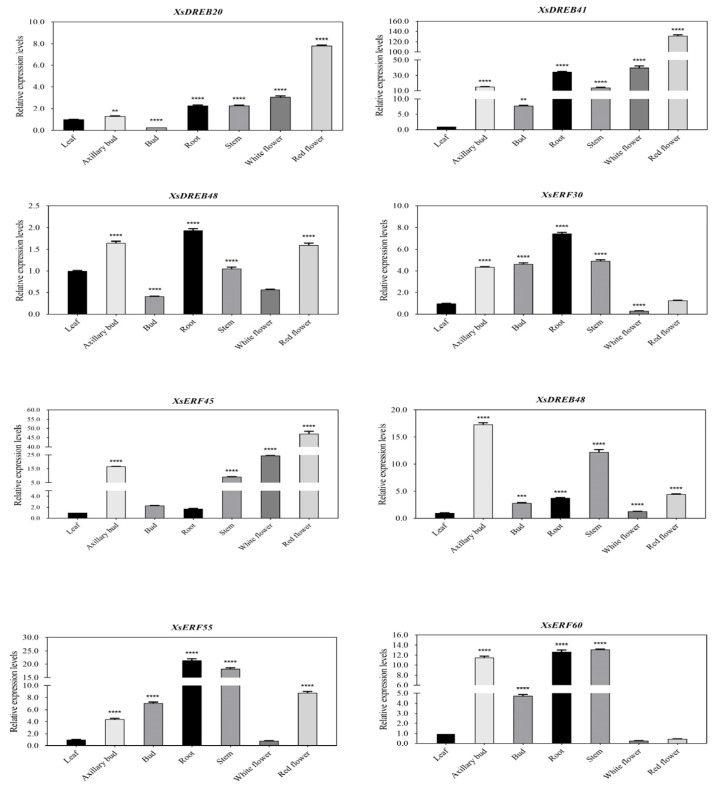
Real-time PCR analysis of eight *XsAP2/ERF* transcription factors in leaves, axillary buds, buds, stems, roots, safflower, and white flowers of mature plants. The *X*-axis is different tissues. The *Y*-axis represents the relative expression levels of transcription factors. The data represent the mean ± SE of three dependent replicates. A *p*-value less than 0.01 (**), 0.001 (***), or 0.0001 (****) indicates statistical significance.

We analyzed the expression levels of *XsAP2/ERF* in seven different tissues (leaves, axillary bud, bud, stem, root, safflower, white flower) of mature plants (unstressed) by RT-qPCR. The expression levels of *XsERF30, 48, 55,* and *60* were significantly different in axillary buds, buds, stems, and roots, among which two genes (*XsDREB41* and *XsERF45*) were highest expressed in safflower and white flowers (Figure 6), and three genes (*XsERF45, 48* and *60*) were highly expressed in axillary buds. *XsDREB41*, *XsERF55,* and *XsERF60* were highly expressed in shoots, roots, and stems. Overall, the expression level of *XsDREB41* was high in all tissues.

**Figure 7:**
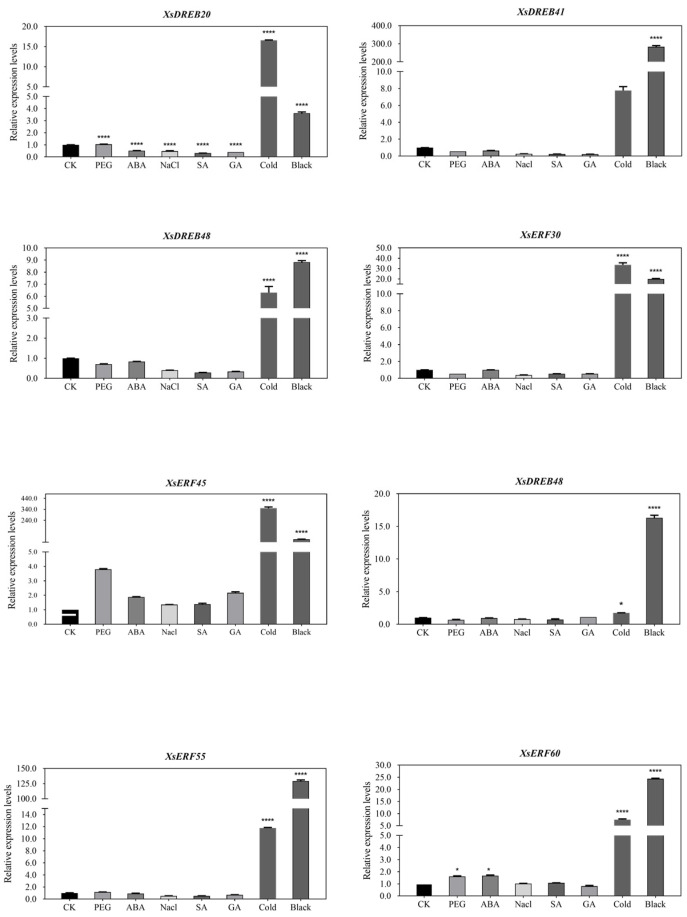
Real-time PCR analysis of 8 *XsAP2/ERF* transcription factors treated for 24 h under PEG, salt, cold, ABA, SA, and GA conditions and 48 h under dark conditions. The *X*-axis is a different treatment. The *Y*-axis represents the relative expression levels of transcription factors. The data represent the mean ±SE of three dependent replicates. A *p*-value less than 0.05 (*), or 0.0001 (****) indicates statistical significance.

To further explore the response of *XsAP2/ERF* genes to abiotic stress conditions (salinity, drought, cold, and dark treatments) and plant hormone induction, we analyzed the expression levels of eight *XsAP2/ERF* genes by RT-qPCR (Figure 7). Interestingly, all tested *XsAP2/ERF* gene family members showed up-regulated expression under cold stress and dark treatment for 24 h. *XsDREB20* and *XsERF60* were up-regulated under PEG treatment for 24 h, *XsERF60* was up-regulated under ABA treatment for 24 h, and *XsDREB20* was down-regulated under all hormones treatment for 24 h. Our abiotic stress response expression analysis results indicate the potential role of AP2/ERF family genes in regulating cold stress and dark stress responses in *X. sorbifolium*.

## 3. Discussion

To date, the AP2/ERF family is prominent in dicotyledons and monocots that play an essential role in plants [10] to protect themselves from environmental stimuli and improve growth and stress tolerance [29,30]. Thus, identifying AP2/ERF TFs could significantly enhance our understanding of the evolution and function of these TFs in different plant species. Based on the release of the other genome sequence data, a previous study reported the identification and functional analysis of the AP2/ERF, which will provide essential clues to further functional characterization of each AP2/ERF TFs.

The AP2/ERF gene family contains 145 members, identified using the PFAM ID ‘PF00847′ in yellow horn. The number of *X. sorbifolium* was close to the number of *A. thaliana* (147) [10], soybean (147) [27], grape (149) [26] but was less than rice (163) [25] and poplar (200) [28]. This is likely because the ERF/DREB subfamily of AP2/ERF TFs is the largest, with 119 members being similar to *A. thaliana* (122), soybean (120), and grapevine (122) but less than rice (145) and poplar (169). Like most plant species, the identified number of AP2/ERF members is determined by the number of ERF/DREB subfamily members [31].

According to the gene structure and evolutionary rate, the AP2/ERF TFs were classified as 54 DREB, 65 ERF, 21 AP2, 4 RAV, and 1 Soloist. Generally, the number of genes in each family was similar as a percentage of the total number of genes in the AP2/ERF superfamily between plant species. The number of genes in each family was similar as a percentage of the total number of genes in the AP2/ERF superfamily among plant species. For example, the ratio of ERF family members is just as high as that of AP2 family members within the AP2/ERF family. The AP2 family has about four times as many genes as the RAV family, which has about four to five times as many genes as the Soloist gene.

The AP2/ERF gene structure analysis indicated that more AP2 family members contained more than two introns. However, most members had few introns in other subfamily members. For example, 85.18% of DREB subfamily members lack introns, and 58.00% of ERF subfamily members have no intron structure. All of the AP2 subfamilies contain introns and exons, and a Soloist gene has six introns. In summary, most ERF and DREB subfamily members in yellow horn possessed no or one intron. In contrast, almost all the AP2 and Soloist subfamilies contained 2–9 introns. The gene and protein structure variance demonstrate a divergence of different subfamilies while highly conserved within subfamilies. Losing introns in ERF subfamily members during evolution may impact their function [30].

Multiple studies indicated that gene duplication events, including segmental duplication, tandem duplication, transposition, and whole-genome duplication, are the primary source of evolution and expansion of the gene families [32,33,34]. The AP2/ERF gene family has expanded due to segmental and tandem duplications, which might play a role in the evolution. We found that 18 pairs of tandem duplication and 27 pairs revealed segmentary repeat events in yellow horn, indicating that these genome duplication events have been recorded as responsible forces for the expansion of the AP2/ERF gene family, which is consistent with previous findings [35]. The number of ERF and DREB genes in different plants varied significantly, proving this point. However, the number of introns in the RAV subfamily genes of *X. sorbifolium* showed less variation, indicating that these genes were highly conserved and shared a common ancestor before separation from the other plants. Moreover, only 32 duplicated gene pairs were found in yellow horn, which was lower than 51 gene pairs of *Arabidopsis*, 41 gene pairs of rice, 90 gene pairs of sunflower, and 76 gene pairs of grapevine. Hence, the low number of gene replication events might be due to the low number of AP2/ERF genes in *X. sorbifolium*.

The results of the collinearity analysis demonstrated that there are 75 pairs of homologous genes between yellow horn and *Arabidopsis*. Still, only 39 pairs of homologous genes were identified between yellow horn and rice. This finding further suggests that the yellow horn had higher homology with *Arabidopsis*. Furthermore, the *XsAP2/ERF* gene clusters or hot regions were mapped to chromosomes LG01, LG03, LG011, and LG012, suggesting the expansion of the *XsAP2/ERF* gene family may have been the result of tandem duplication and segmental duplications. The Ka/Ks values of all gene pairs in the *XsAP2/ERF* members were less than 1, indicating that these genes may be affected by purification selection instead of positive selection during their evolution.

The evolutionary relationships among AP2/ERF family proteins of yellow horn and *Arabidopsis* were used to build a phylogenetic tree, indicating that the AP2/ERF family was clustered into four subfamilies that were homologous between *X. sorbifolium* and *Arabidopsis* [10,28].

Plant transcription factors generally have four functional domains, namely DNA binding region, oligomeric site, transcription control region, and nuclear localization signal [10], which are essential in regulating gene expression and various biological processes [14]. Identifying conserved motif structures in phylogenetic clades helps to understand the variation and evolution of gene function [36]. A different subfamily of the AP2/ERF gene family has undergone a specific expansion/contraction during the development of other plants [37]. Most genes in the same class usually share similar conserved motifs and gene structures. A total of 50 AP2/ERF protein motifs have been characterized in *A.thaliana* [10]. Combined with phylogenetic trees, we found 25 AP2/ERF protein motifs using MEME and named motifs 1–25. Among them, motif one was shared by all members. The AP2/ERF superfamily has high conservation [38], while different *XsAP2/ERF* proteins have other conserved motifs. In general, the highly similar gene structure and conserved motif composition of *XsAP2/ERF* genes within the same subgroup strongly support the results of the phylogenetic analysis [39].

In addition, we tested the expression of eight *XsAP2/ERF* genes in various tissues, including leaves, axillary buds, buds, stems, roots, red flowers, and white flowers by RT-qPCR. The transcription factors were highly expressed in all tissues, with *XsDREB41* showing the highest expression levels. However, leaves showed lower levels of expression for these factors. Previous reports have demonstrated that AP2/ERF family members are widely expressed in various tissues and organs, regulating plant growth and development [40,41]. Many studies have demonstrated that multiple family members of TFs have contributed to controlling a wide range of defense responses and adapting to various changing environmental conditions in different plants.

The mechanism of these inducible genes is unknown, and this lack of understanding extends to the expression profiles of the *XsAP2/ERF* genes under abiotic stress. By analyzing the gene expression patterns of *XsAP2/ERF* genes under low-temperature stress, we found that the transcription levels of most co-expressed genes were up-regulated. The DREB TFs have been widely identified in various species like rice [42], soybean [43], maize [35], tomato [44], and tamarix [45]. These transcription factors are vital in regulating plant stress responses, especially A1 and A2 DREB subfamily members [46,47]. *XsDREB32 and XsDREB33*, which belong to the DREB A1 subgroup, were significantly up-regulated under cold stress in our study. Meanwhile, RAP2.1 overexpression also leads to enhanced sensitivity to abiotic stresses, such as cold and drought stresses [48]. Three DREBs and five ERFs were up-regulated under cold stress conditions according to RT-qPCR analysis. These findings are consistent with past reports of the AP2/ERF transcription factors regulated by cold stress. Overall, these results provide valuable information for a better understanding of the function of AP2/ERF genes in yellow horn.

The cis-acting elements in the regulatory sequences upstream of the gene can influence the response to stress and hormones, growth, and development. They perform essential functions, such as integrating stress signals and controlling downstream stress responses under various abiotic stress treatments [47]. Promoter analysis of *XsAP2/ERF* members revealed that almost all *XsAP2/ERF* members contain conserved ABA-response elements. In addition, a large number of stress-related cis-elements were identified, including low-temperature responsive elements (LTRs) [48], drought-responsive elements (MYBs, MYCs) [49], light response elements, and dehydration-responsive elements (DREs). These genes might play critical functions in light signaling, hormone, and stress response. Our results showed high expression levels of four genes (*XsDREB20/32/33/48*) by RNA-seq, which have abundant stress-related cis-acting elements under abiotic stress. Meanwhile, eight *XsAP2/ERF* genes that possessed LTR and light-responsive elements were significantly up-regulated under the treatment of cold and dark stress considerably by RT-qPCR.

In sum, it was suggested that the expression of *XsAP2/ERF* genes in yellow horn is regulated by cis-elements linked with hormone signal transduction and abiotic stress tolerance.

## 4. Materials and Methods

### 4.1. Identification and Classification

Yellow horn (*Xanthoceras sorbifolia*) genome files were downloaded from the GigaDBdata (http://gigadb.org/dataset/view/id/100606, accessed on 7 June 2021). The AP2/ERF protein sequences of *Arabidopsis thaliana* and *Oryza sativa* were retrieved from PlantTFDB (http://planttfdb.gao-lab.org/, accessed on 7 June 2021). The obtained proteins sequence of *X. sorbifolium* were used as query sequences in the BLAST software (the primary local alignment search tool) to screen members of the AP2/ERF gene family. The Hidden Markov Model (HMM) profiles of AP2 domains (PF00847) were obtained from the Pfam and used to HMM search against the yellow horn genomic database by HMMER3.0. The default parameters were used, and the cutoff value was set to 0.001. Then, the *XsAP2/ERF* genes were rechecked using the NCBI CDD (Conserved Domains) database (https://www.NCBI.nlm.nih.gov/cdd, accessed on 7 June 2021) to remove non-family members and repetitive sequences.

### 4.2. Phylogenetic Analysis

The ClustalW program was used to align multiple candidate AP2/ERF genes with default parameters [50]. The three phylogenetic trees were produced using the neighbor-joining method in MEGA 6.0 software [51]. The bootstrap values were calculated based on 1000 replicates for node support.

### 4.3. Gene Structure and Conserved Motif

The Multiple Em for Motif Elicitation (MEME) 5.0.5 online tool (https://meme-suite.org/meme/tools/meme, accessed on 7 June 2021) [52] was used to identify conserved protein motifs with the following parameters: zero or one occurrence per sequence the maximum number of motifs, 25; optimum motif widths ranging from 6 to 50 residues. The exon-intron structure of *XsAP2/ERFs* was recorded from the gene structure Display Server (GSDS 2.0) [53]. The phylogenetic trees, conserved motifs, and gene structure of *XsAP2/ERF* genes were shown by TBtools software [54].

### 4.4. Chromosome Distribution and Duplication

The chromosomal localization information of the AP2/ERF genes in yellow horn was retrieved from the reference genome database. We used the TBtools software to visualize the distribution of genes on chromosomes, with Multiple collinear Scanning toolkits (MCScanX) [55] used to analyze gene duplication events.

The syntenic relationships between *XsAP2/ERF* and AP2/ERF genes from *Arabidopsis thaliana* and *Oryza sativa* were generated using Dual Synteny Plotter software. Nonsynonymous (Ka), synonymous (Ks) substitutions and the Ka/Ks ratio were calculated in a pairwise comparison by KaKs_Calculator Version 2.0 [56].

### 4.5. Cis-Acting Elements Analysis of AP2/ERF Superfamily Genes

The 2000 bp sequences upstream of the *XsAP2/ERF* genes transcriptional start site were submitted to the online site PlantCARE to predict their cis-acting elements. (http://bioinformatics.psb.ugent.be/webtools/plantcare/html/, accessed on 7 June 2021).

### 4.6. Plant Materials and Stress Treatments

#### 4.6.1. Transcriptome Analysis of AP2/ERF

Transcriptome data under salt, alkali, and low-temperature stress were downloaded from the NCBI short read archive database (SRA database, https://www.ncbi.nlm.nih.gov, accession number SRX7830132—SRX7830143 and SRX8911738—SRX8911752). We used to map all transcriptome data to the *X. sorbifolium* reference genome [57]. *XsAP2/ERF* gene expressional values were evaluated using the Cufflinks software previously described [58]. TBtools drew heat maps of salt, alkali, and low-temperature stress.

#### 4.6.2. Real-Time Quantitative PCR (RT-qPCR) of XsAP2/ERF

Seedlings were cultivated in the greenhouse of the College of Forestry, Shanxi Agricultural University (22–25 °C, 16 h light, 8 h darkness) for three months. The plant material was selected for abiotic and hormone treatments for 24 h: 100 μM gibberellin A3 (GA3), 100 μM of abscisic acid (ABA), 200 μM salicylic acid (SA), 10% polyethylene glycol 6000 (PEG6000), 100 mM sodium chloride (NaCl), 4 °C low temperature. Dark control seedlings were kept in darkness. The leaves of the plant were harvested 24 h after exposure to the abiotic factors and hormone treatments. In experimental fields, samples of different tissues were collected from triennial plants at the flowering stage. All models were frozen immediately in liquid nitrogen and stored at −80 °C before RNA extraction.

RT-qPCR experiments were performed on an ABI 7500 system (Applied Biosystems, Carlsbad, CA, USA), and the same RNA samples were used. UBC2 (EVM0006862) was used as an internal control. The specific primer pairs of the selected genes were designed using Primer3 (https://bioinfo.ut.ee/primer3-0.4.0/, accessed on 7 June 2021) (Table S3). The relative expression levels of the genes were measured using the comparative 2^−ΔΔCt^ method, and each sample was repeated three times.

## 5. Conclusions

This study comprehensively analyzed the AP2/ERF TFs in yellow horn. To this end, 145 AP2/ERF family genes were identified as potential candidates and were subsequently divided and analyzed in detail. Their evolutionary characteristics, expression patterns in different tissues, and their response to salt, alkali, and low-temperature stress were studied. The expression of transcription factors was generally consistent with the correlated gene expression patterns in leaves. Taken together, these findings promoted a further understanding of the biological function of the AP2/ERF superfamily members. They helped to choose the potential better AP2/ERF transcription factors in regulating abiotic stress responses in the yellow horn.

## Data Availability

The raw RNA-seq data are available in the National Center for Biotechnology Information (NCBI) under SRA accession number: SRP251348.

## Supplementary Materials

The following are available online at https://www.mdpi.com/article/10.3390/ijms232314991/s1, Figure S1: Phylogenetic relationships, gene structure, and motif analysis of conserved protein motifs in AP2/ERF gene from yellow horn, Figure S2: Cis-acting elements and phylogenetic tree of AP2/ERF gene promoter region in yellow horn, Figure S3: Expression profile of *XsAP2/ERF* gene under salt, alkali, and low-temperature stress levels of yellow horn. Table S1: List of *XsAP2/ERF* genes identified, Table S2: Gene duplication analysis of *XsAP2/ERF* genes, Table S3: q-PCR primers.
