# Supplementary material for: Identification and Characterization of AP2/ERF Transcription Factors in Yellow Horn
Source: Int J Mol Sci. 2022 Nov 30;23(23):14991. doi: 10.3390/ijms232314991 (PMC9741253; doi:10.3390/ijms232314991)
Supplement: Supplementary file 1 [file ijms-23-14991-s001.zip › ijms-2016902-SI.pdf]

Table S1: List of *XsAP2/ERF* genes identified

Gene ID of *X.sorbifolia*	Gene name	Theoretical pI	Molecular weight (kDa)
EVM0000269	XsAP2-8	6.53	64.20
EVM0000338	XsDREB7	4.61	21.80
EVM0000408	XsERF9	5.22	30.44
EVM0000610	XsDREB11	4.95	21.19
EVM0000749	XsDREB4	8.98	48.39
EVM0001018	XsERF34	8.48	78.96
EVM0001185	XsDREB54	6.03	32.36
EVM0001246	XsDREB42	5.21	18.41
EVM0001283	XsERF26	6.66	25.26
EVM0001332	XsAP2-15	6.65	52.25
EVM0001546	XsAP2-6	6.08	60.35
EVM0001672	XsRAV3	9.16	39.87
EVM0001843	XsERF54	7.88	24.86
EVM0001992	XsDREB39	6.03	32.36
EVM0002147	XsDREB37	5.15	24.71
EVM0002171	XsERF20	5.43	25.35
EVM0002361	XsERF31	9.62	18.40
EVM0002413	XsAP2-11	6.53	76.02
EVM0002503	XsERF1	9.36	27.82
EVM0002591	XsDREB34	7.71	22.75
EVM0002609	XsAP2-20	8.47	50.71
EVM0002707	XsDREB35	5.48	26.22
EVM0002813	XsERF57	4.84	41.05
EVM0002827	XsAP2-2	6.68	43.57
EVM0002907	XsDREB5	5.17	30.13
EVM0003202	XsDREB30	5.53	28.38
EVM0003557	XsRAV4	9.13	27.46
EVM0003612	XsAP2-3	6.49	83.67
EVM0004012	XsSoloist1	9.64	26.76
EVM0004174	XsDREB41	6.25	25.15
EVM0004440	XsDREB24	5.28	28.26
EVM0004588	XsAP2-21	6.67	47.86
EVM0004634	XsDREB13	5.46	20.88
EVM0004982	XsDREB47	6.61	38.78
EVM0005327	XsERF25	9.05	22.89
EVM0005328	XsAP2-5	6.65	54.92
EVM0005440	XsERF2	5	36.34
EVM0005604	XsERF51	10.38	11.86
EVM0005721	XsERF59	9.33	31.39
EVM0005738	XsDREB33	5.12	24.93

EVM0005833	XsERF43	6.71	24.25
EVM0005850	XsDREB10	4.91	19.38
EVM0005981	XsDREB32	5.04	25.14
EVM0006148	XsDREB2	6.93	45.53
EVM0006523	XsDREB21	5.24	27.17
EVM0006627	XsERF42	9.77	32.21
EVM0006642	XsERF53	7.88	24.86
EVM0006812	XsDREB1	5.94	20.23
EVM0006858	XsERF17	7.89	24.68
EVM0007002	XsERF33	8.45	23.94
EVM0007008	XsDREB45	9.7	26.40
EVM0007314	XsAP2-14	9.31	43.55
EVM0007352	XsERF27	8.82	15.12
EVM0007555	XsERF50	4.78	41.65
EVM0007638	XsERF6	4.9	27.89
EVM0007694	XsERF30	9.38	25.18
EVM0007713	XsAP2-16	7.69	55.95
EVM0007868	XsERF18	6.53	30.63
EVM0008062	XsDREB9	5.77	19.56
EVM0008304	XsERF61	5.78	27.86
EVM0008896	XsERF47	4.99	37.09
EVM0009029	XsDREB28	8	36.01
EVM0009634	XsERF52	8.94	21.05
EVM0009984	XsDREB43	4.95	63.51
EVM0010098	XsERF15	5.33	22.29
EVM0010202	XsDREB51	6.82	37.44
EVM0010292	XsERF12	6.92	29.66
EVM0010492	XsDREB15	5.65	18.28
EVM0010587	XsERF28	5.94	47.12
EVM0010818	XsERF36	4.6	31.84
EVM0011042	XsERF22	8.01	15.62
EVM0011203	XsDREB49	6.61	38.76
EVM0011301	XsERF46	8.71	20.38
EVM0011691	XsERF44	9.7	32.33
EVM0012018	XsDREB20	9.14	18.78
EVM0012075	XsAP2-17	5.3	44.54
EVM0012620	XsDREB18	5.49	23.89
EVM0012792	XsERF23	5.92	30.11
EVM0012840	XsERF35	7.75	34.96
EVM0012901	XsERF56	6.86	23.92
EVM0012951	XsDREB12	4.9	20.91
EVM0013275	XsERF11	5.65	29.68
EVM0013598	XsAP2-10	6.38	57.57
EVM0013648	XsDREB14	5.65	18.26

EVM0013998	XsDREB3	5.25	29.64
EVM0014170	XsDREB48	4.92	32.84
EVM0014310	XsDREB38	6.03	32.36
EVM0014579	XsRAV2	6.73	42.56
EVM0014774	XsERF60	9.26	21.86
EVM0014879	XsAP2-7	9.3	43.69
EVM0015104	XsRAV1	6.42	40.83
EVM0015189	XsDREB8	8.88	17.44
EVM0015396	XsERF45	6.18	43.51
EVM0015403	XsERF62	9.47	24.56
EVM0015700	XsERF55	7.67	28.81
EVM0016183	XsAP2-9	8.49	38.32
EVM0016535	XsDREB31	5.53	28.39
EVM0016628	XsDREB44	7.74	24.18
EVM0017076	XsDREB46	5.39	21.78
EVM0017184	XsDREB36	5.03	25.01
EVM0017351	XsERF24	5.56	42.46
EVM0017404	XsERF37	4.73	44.13
EVM0017598	XsERF48	7.68	27.33
EVM0017711	XsERF21	8.8	18.734
EVM0017944	XsERF19	6.97	30.65
EVM0018311	XsDREB19	6.67	40.05
EVM0018364	XsDREB6	4.98	25.69
EVM0018575	XsAP2-19	7.69	55.95
EVM0018636	XsERF63	7.77	34.32
EVM0018895	XsERF32	5.04	29.89
EVM0019345	XsDREB50	8	35.99
EVM0019371	XsERF14	6.35	26.72
EVM0019480	XsERF16	6.97	24.53
EVM0019842	XsAP2-18	8.11	54.19
EVM0020103	XsERF29	5.43	32.40
EVM0020368	XsERF3	5.26	34.72
EVM0020672	XsDREB52	5.24	27.20
EVM0020690	XsERF4	8.4	32.91
EVM0020885	XsERF65	5.06	33.73
EVM0020978	XsDREB23	5.06	27.06
EVM0021093	XsAP2-4	6.77	59.29
EVM0021692	XsAP2-12	6.02	56.78
EVM0021741	XsERF8	5.64	27.58
EVM0022247	XsERF40	9.32	20.99
EVM0022422	XsAP2-1	6.38	76.03
EVM0022551	XsERF5	8.96	32.87
EVM0022696	XsDREB27	8.89	19.47
EVM0022808	XsERF64	9.7	32.33

EVM0022902	XsERF7	5.65	27.56
EVM0023024	XsDREB53	8.89	19.47
EVM0023072	XsAP2-13	8.24	41.30
EVM0023237	XsDREB16	5.94	18.35
EVM0023269	XsDREB17	6.83	18.26
EVM0023304	XsERF49	5.22	38.06
EVM0023345	XsDREB40	5.77	25.88
EVM0023356	XsERF39	4.73	44.88
EVM0023623	XsERF13	6.13	29.73
EVM0023930	XsDREB25	5.78	43.39
EVM0024091	XsERF41	6.34	33.12
EVM0024122	XsDREB26	8.89	19.47
EVM0024151	XsERF38	5.85	46.83
EVM0024324	XsDREB29	5.97	18.21
EVM0024400	XsDREB22	5.58	24.88
EVM0024549	XsERF10	9.47	24.56
EVM0024593	XsERF58	5.06	33.73

Table S2: Gene duplication analysis of *XsAP2/ERF* genes

Duplicated gene pairs	Ka	Ks	Ka_Ks	Length	S-sites	N-sites	cN	cS	pN	pS
XsERF6-vs-XsERF20	0.24	2.8	0.09	648	142.17	505.83	104.92	104.08	0.21	0.73
XsDREB26-vs-XsERF20	0.37	3.5	0.11	459	106.58	352.42	102.83	79.17	0.29	0.74
XsAP2-11-vs-XsAP2-1	0.21	1.7	0.12	1974	436.75	1537.25	285.58	295.42	0.19	0.68
XsDREB33-vs-XsDREB42	0.49	3.7	0.13	495	118.75	376.25	134.58	88.42	0.36	0.74
XsERF7-vs-XsERF21	0.34	2.3	0.15	456	106.42	349.58	94.83	76.17	0.27	0.72
XsDREB23-vs-XsDREB24	0.33	2.2	0.15	657	155.58	501.42	132.58	110.42	0.26	0.71
XsERF37-vs-XsERF58	0.3	1.8	0.17	882	196.58	685.42	168.08	133.92	0.25	0.68
XsERF30-vs-XsERF60	0.3	1.4	0.22	579	141.08	437.92	108.17	88.83	0.25	0.63
XsAP2-5-vs-XsAP2-16	0.37	1.3	0.28	1347	313.00	1034.00	300.42	193.58	0.29	0.62
XsDREB5-vs-XsDREB23	0.34	1.1	0.32	711	166.25	544.75	149.00	94.00	0.27	0.57
XsERF2-vs-XsERF23	0.63	1.8	0.36	714	165.75	548.25	234.67	112.33	0.43	0.68

Table S3: q-PCR primers

Primer name	Sequence 5' to 3'
UBC2-FP	ATTGGAGATGGAACTGTA
UBC2-RP	TTCAACTGGTAGATACGA
XsERF48-FP	CTTACGACCGAGCCGCTTAT
XsERF48-RP	CGACGTAATCCGAACTGGGT
XsDREB41-FP	ATAGTTTGTCGCGTCTGGGG
XsDREB41-RP	TTCTTGTTCGGCTCCCTCAC
XsDREB20-FP	TCTGGCTTGGCTCTTACACC
XsDREB20-RP	TGCGTATAGAAGCGGCTGAC
XsERF55-FP	ACGGAAACAGTGACGGAGAC
XsERF55-RP	ACATAACCGTCAGCCGTGAG
XsDREB48-FP	GCCGCATAGAGGGAAAAGGT
XsDREB48-RP	GCAATCCTCCACCTCGGAAA
XsERF60-FP	GGTTCAGGGGTGTCAGGAAG
XsERF60-RP	TTGATTTCATCCGGGGTGGG
XsERF30-FP	GATGATCGATTCCTCGCCGT
XsERF30-RP	GCCGAACCAAAAACTCCACC
XsERF45-FP	ACCGACTCACTTCGCCATTT
XsERF45-RP	TTTTCGCCATCGGGACATCA

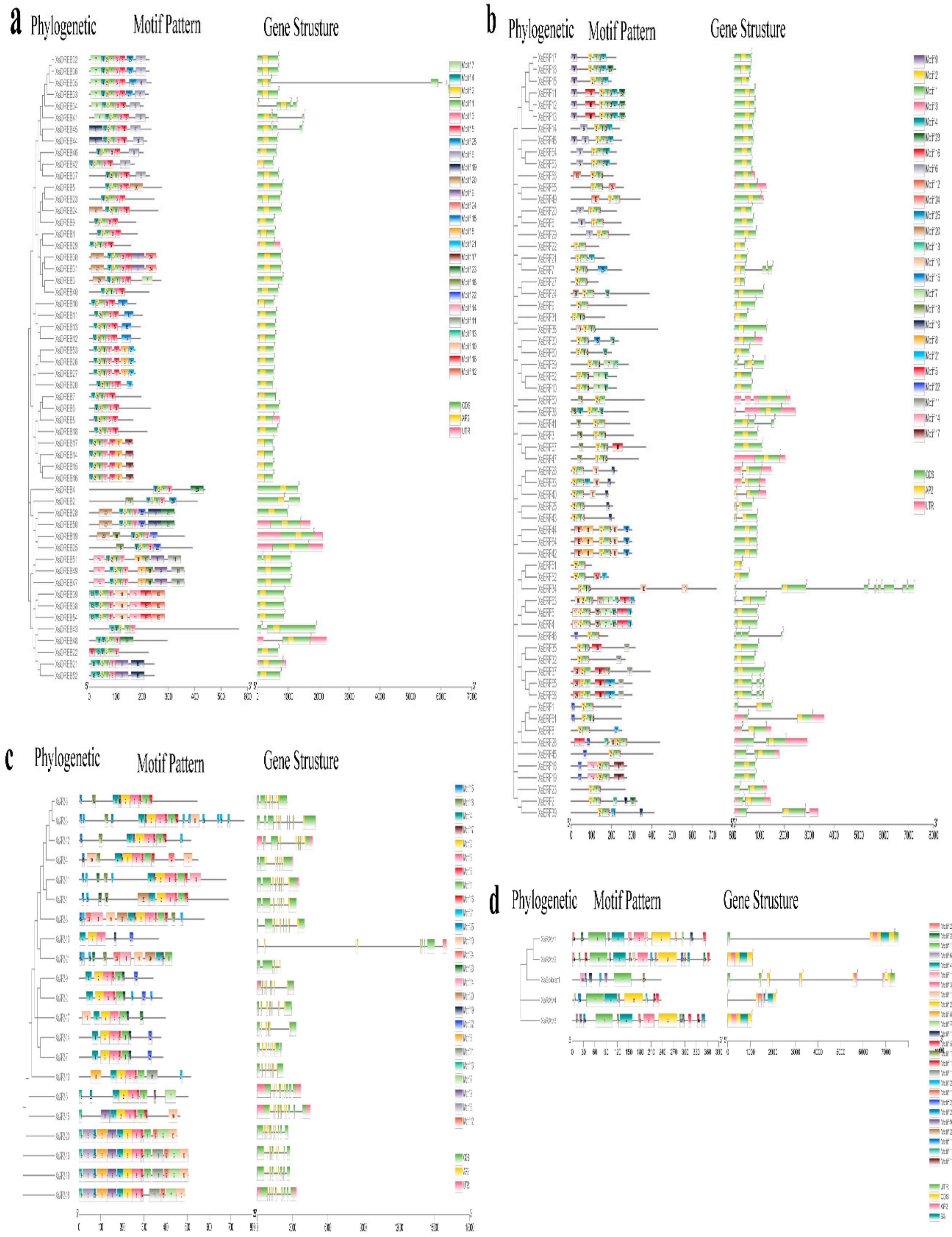

Figure S1. Phylogenetic relationships, gene structure, and motif analysis of conserved protein motifs in AP2/ERF gene from yellow horn. (a) Phylogenetic relationships, gene structure, and conserved motifs in the DREB subfamily, (b) phylogenetic relationships, gene structure, conserved motifs in the ERF subfamily, (c) phylogenetic relationships, gene structure, and conserved motifs in the AP2 subfamily, (d) phylogenetic relationships, gene structure, and conserved motifs in the RAV and Soloist subfamily. Clustal W and MEGA 6 were used to align the complete amino acid sequence and construct the phylogenetic tree. The untranslated region (UTR), exons, and introns are represented by green boxes, yellow boxes, and gray lines, respectively.

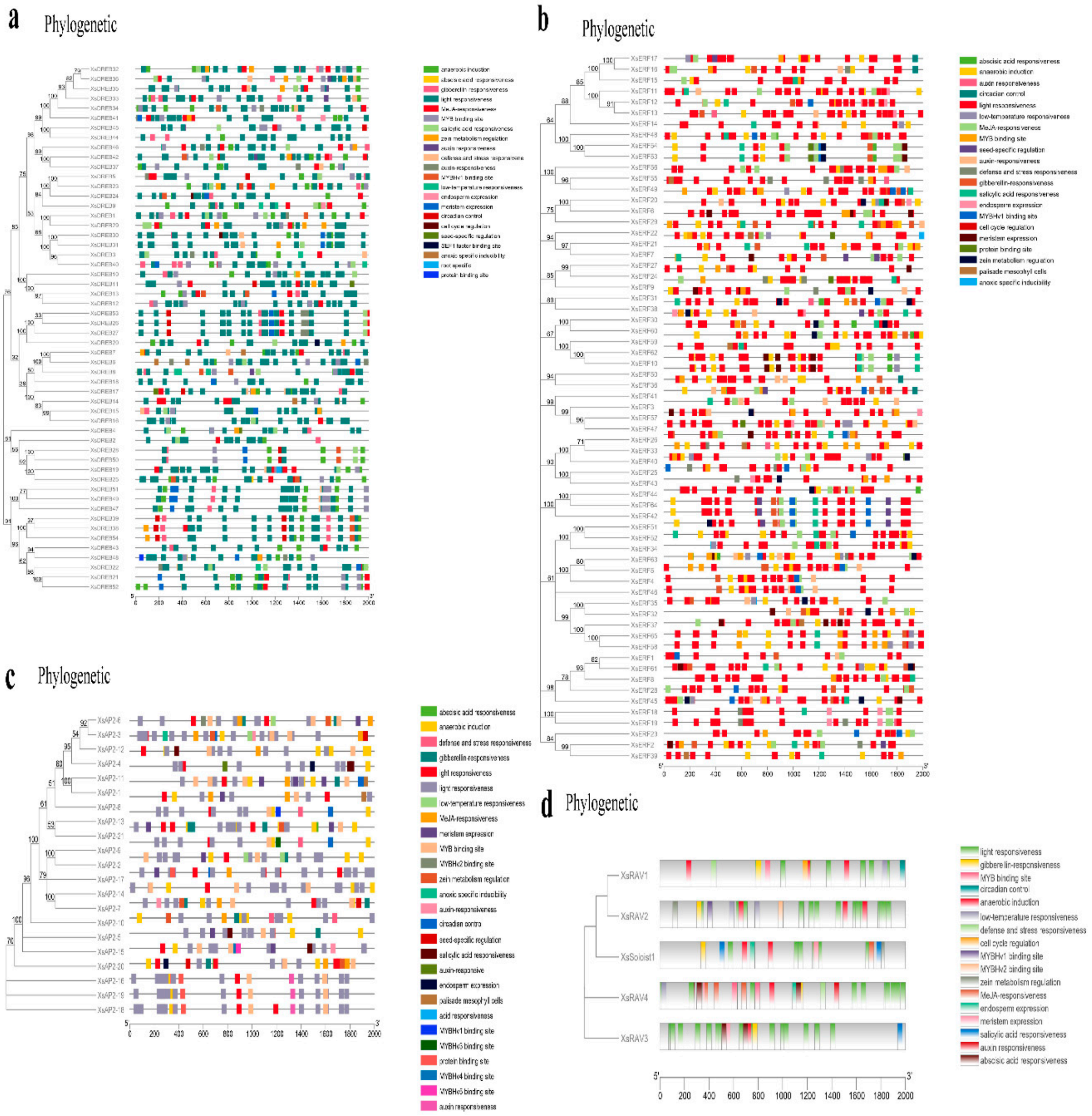

Figure S2. Cis-acting elements and phylogenetic tree of AP2/ERF gene promoter region in yellow horn. (a) Cis-acting elements and phylogenetic tree of the DREB subfamily, (b) cis-acting elements and phylogenetic tree of the ERF subfamily, (c) cis-acting elements and phylogenetic tree of the AP2 subfamily, (d) cis-acting elements and phylogenetic tree of the RAV and Soloist subfamily. Different colors in the boxes represent the cis-acting elements.

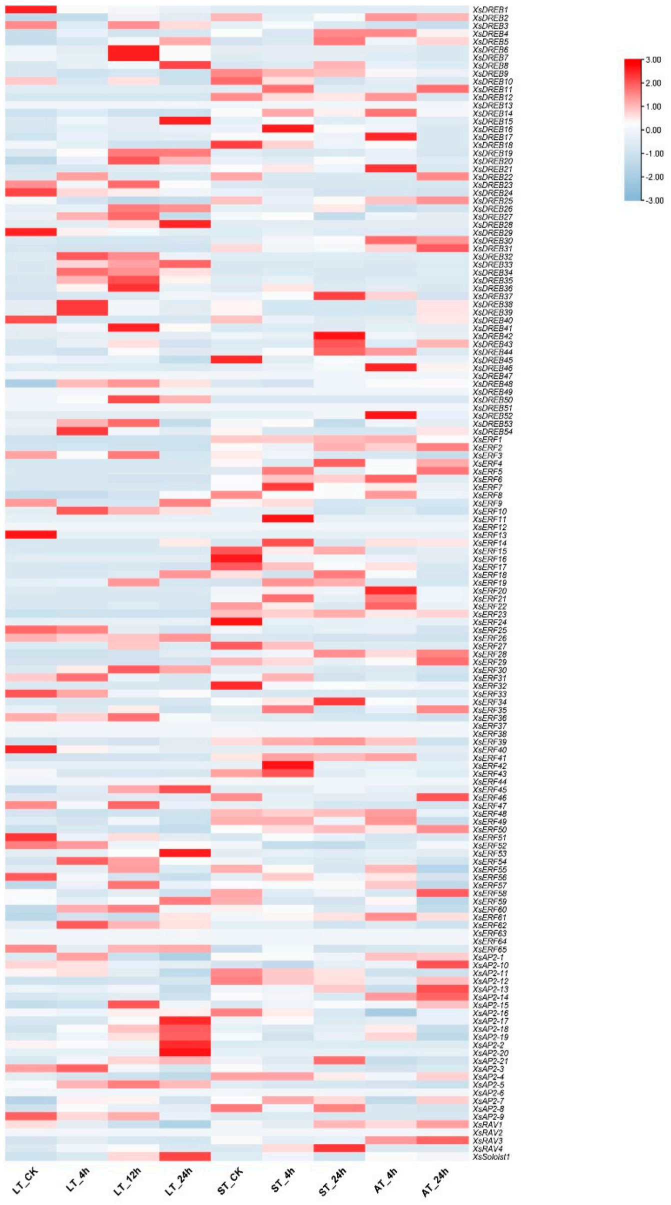

Figure S3. Expression profile of *XsAP2/ERF* gene under salt, alkali and low-temperature stress levels of yellow horn TBtools created heat maps based on transformed data. For each row, the expression pattern is presented as a blue/white/red/coded heat map after a z-score standardized transformation, with red indicating up-regulated expression and blue indicating down-regulated expression.
